# Preparing Medical Students for Sideline Sports Care in Underserved Communities: A Comparison of Simulation and Lecture-Based Training

**DOI:** 10.7759/cureus.86208

**Published:** 2025-06-17

**Authors:** Alqasim Elnaggar, David Abdelnour, Ammar Chauhdri, Candice Miller, Rumyah Rafique, Jack Mao, Ashley Frei, Sara Naessig, Julian D Johnson, Marley Sternberg, Audrey Millar, Tannor Court, Fong Nham, Ahmed Daher, Andrew Gupta, Joshua Gatz, Radomir Dimovski, James Alexander, Andreea Geamanu, Rahul Vaidya

**Affiliations:** 1 Department of Orthopaedic Surgery, Wayne State University School of Medicine, Detroit, USA; 2 Department of Orthopaedic Surgery, Wayne State University Detroit Medical Center, Detroit, USA; 3 Department of Family Medicine, Wayne State University School of Medicine, Detroit, USA; 4 Athletics, Detroit Public Schools Community District, Detroit, USA

**Keywords:** community-based medical education, inreach, simulation-based training, sports injury management, underserved communities

## Abstract

Introduction: Access to sideline medical care is limited in many public-school athletic programs, especially in underserved urban areas. The Detroit Public School Health Corps (DPSHC) was founded in response to the tragic death of a Detroit public school basketball player who suffered a cardiac arrest during a game, underscoring the critical need for timely and effective sideline emergency care. DPSHC is a student-led inreach initiative targeting local healthcare gaps by providing equity-driven medical training in nearby schools. This study tests the hypothesis that simulation-based training will be more effective than traditional didactic training in improving a student volunteer’s clinical preparedness, knowledge retention, and confidence in sideline medical roles. The primary objectives are to compare confidence and knowledge gains between the two training methods, while a secondary objective evaluates the perceived impact of resident physician involvement during simulation.

Methods: Forty-seven medical student volunteers completed either lecture-based (n=16) or simulation-based (n=31) training. Both groups completed identical pre- and post-training surveys assessing confidence in managing sports injuries and clinical knowledge. Paired t-tests analyzed the data to evaluate within-group improvements from before to after a training session, while independent t-tests compared the degree of improvement between training formats.

Results: Both groups showed a significant improvement in confidence (p < 0.001). The simulation group showed greater confidence gains than the lecture group (33.06% vs. 24.53%, p = 0.02). Knowledge scores improved more in the simulation group (62.1% vs. 31.25%), though not significantly (p = 0.153). Resident physician involvement was rated invaluable by 25 (80.6%) of the simulation participants.

Conclusion: Simulation-based training enhanced medical student volunteers’ confidence more than lecture-based methods in sideline care roles within an inreach program addressing healthcare disparities. These findings support the expansion of simulation-based learning as a core component of equity-driven, community-embedded medical education.

## Introduction

Equitable access to sideline healthcare remains an unmet need in many public-school athletic programs, particularly in underserved urban communities [1,2]. The Detroit Public School Health Corps (DPSHC) was founded in response to a tragic event in which a Detroit high school basketball player suffered a fatal cardiac arrest during a game. This loss underscored the critical importance of timely and effective sideline care, motivating efforts to improve emergency response for the more than 20,000 student athletes served annually by the program. DPSHC deploys trained medical student volunteers, known as health leaders (HLs), to provide immediate first aid and triage at high school sports events in neighboring public schools.

While medical institutions often emphasize outreach to distant or global populations, significant healthcare disparities persist and must be addressed within the immediate environments surrounding these institutions. We refer to this concept as inreach: targeted, equity-driven initiatives that confront structural healthcare gaps right in our front yards and local communities, often overlooked due to their proximity. DPSHC exemplifies this model by expanding healthcare access through local volunteer engagement.

This study tests the hypothesis that simulation-based training will be more effective than traditional didactic training in improving a HLs’ clinical preparedness, knowledge retention, and confidence in sideline healthcare roles. The primary objectives compare confidence and knowledge gains between the two training methods, while a secondary objective evaluates the perceived impact of resident physician involvement during simulation-based training. These aims provide a focused framework for evaluating educational strategies within this unique initiative. Through this inreach-based model, DPSHC not only improves healthcare access but also offers medical students valuable early clinical experiences within their own community. However, providing adequate care in unpredictable sideline settings requires more than lecture-based instruction. Transitioning from academic knowledge to practical competence can be challenging for students, especially in high-pressure environments where confidence and clinical decision-making are critical. Consequently, training models prioritizing experiential learning are increasingly integrated into undergraduate medical education [3-7].

Simulation-based medical education (SBME) has become widely implemented to bridge the gap between theory and practice. Compared to traditional didactic methods, simulation offers structured, repeatable, and immersive learning experiences that promote skill development, psychological preparedness, and learner engagement [3,4,8,9]. As its use expands, SBME is being applied across many specialties, from acute care and surgical skills to orthopedics and pediatrics [7,9,10]. Its ability to improve learner satisfaction and motivation has also been noted, making it a compelling supplement to lecture-based education [6,11].

Several studies have further explored how SBME compares to other instructional formats. Meta-analyses and controlled trials suggest that simulation and flipped-classroom models frequently outperform lectures in promoting clinical competence, knowledge retention, and communication skills [11-14]. These findings have contributed to SBME’s growing role as a supplement to traditional education or a preferred alternative in some areas. This is particularly relevant where practical skills and real-time decision-making are essential, such as in emergency response or procedural specialties. Furthermore, the ability of simulation to offer safe, repeatable practice environments has made it an appealing tool for early learners who may otherwise have limited access to clinical exposure.

Despite this growing body of literature, the application of SBME to student-led, volunteer-based initiatives, particularly those framed as inreach efforts such as DPSHC, remains underexplored. By assessing changes in knowledge and self-reported confidence, we aim to contribute meaningful data to the conversation around optimizing training for early clinical engagement in underserved settings. In doing so, we intend to inform the design of scalable, resource-conscious educational interventions, better equipping students to serve safely and effectively in high-stakes community settings. Additionally, this work may serve as a model for other institutions seeking to integrate inreach strategies into their service-learning frameworks.

## Materials and methods

This study was conducted within the DPSHC program, which places medical student HLs at public school sports events to serve as sideline medics. Consent for treatment and open access publication was obtained or waived by all participants in this study. Wayne State University School of Medicine issued approval (Approval number: 24-11-7337). The primary aim was to assess the effectiveness of two distinct training formats, traditional didactic lecture-based training and an updated simulation-based training model, in preparing HLs for in-game medical responsibilities. A mixed-methods approach was used to compare outcomes, including knowledge acquisition, confidence, and overall preparedness. Participants with no prior training in sideline sports coverage were enrolled voluntarily, from the Wayne State University School of Medicine, as the training sessions pertained to a season-long commitment of sideline coverage with the DPSHC.

The lecture-based cohort included 16 participants, while the simulation-based cohort included 31 participants, resulting in an imbalance in group sizes. Random assignment to training groups was not feasible because participation was voluntary and part of an ongoing service-based initiative. Restricting or randomly assigning students to specific training formats would have been unethical and impractical given the program’s operational needs and growing demand. The difference in group sizes reflects natural program growth over time, with more students joining the initiative by the spring training.

Training format 1: Lecture-based instruction (Fall season)

During the fall sports season, HLs and general volunteers participated in a lecture-based training program, which took place on September 4th, 2024. This session was led by experienced executive board members (presidents, vice-presidents, and founders of DPSHC). Training content focused on key areas including basic medical intervention techniques, sideline injury identification, triage principles, and communication protocols. These sessions were delivered in a classroom setting and were primarily lecture-based, with the only practical, hands-on component being a 30-minute taping demonstration at the end. A pre-training and post-training survey was administered to assess changes in participants’ self-reported confidence levels and clinical knowledge relevant to managing sports injuries.

Training format 2: Simulation-based instruction (Spring season)

In response to participant feedback, the training for the spring sports season, which took place on April 16th, 2025, was redesigned to incorporate a more interactive and experiential learning model. In collaboration with residents and attending physicians from Detroit Medical Center Orthopedics and Sports Medicine, an updated 1.5-hour training session was developed.

The revised training consisted of rotating 30-minute stations between rooms dedicated to sports that volunteers were expected to cover during the season, including track and field, soccer, and baseball/softball. The HLs participated in a 10-minute didactic session preceding an immersive simulation-based exercise at each station. Student actors portrayed injured athletes in an improvisational manner, presenting a range of common sports-related injuries. We tasked the HLs with responding to these mock scenarios as they would in real-life situations, including performing assessments and initiating appropriate interventions such as diagnostic tests like the Lachman, anterior drawer, posterior drawer, McMurray, varus stress, and valgus stress tests. They were also evaluated on their ability to identify and manage potential concussions by conducting a neurological physical exam. Supervising physicians provided real-time feedback and instruction throughout the simulations. Finally, the HLs were instructed to deliver an oral presentation to the physician as they would in the field when an injury necessitates an escalation of care. They were directed to utilize an SBAR (Situation, Background, Assessment, Recommendation) framework, a validated method to ensure safe and effective handoffs to emergency medical service personnel or attending physicians [15].

As with the initial training format, pre- and post-training surveys were administered to assess changes in confidence, clinical decision-making ability, and perceived preparedness. Survey items were standardized across both training formats to allow for direct comparison.

Survey design and outcome measures

To evaluate the effectiveness of the two training formats, we administered a standardized pre- and post-training survey to HLs in both the fall (lecture-based/didactic) and spring (simulation-based) cohorts. The survey was designed to assess two primary outcomes: self-reported confidence and clinical knowledge related to sideline management of common sports injuries. The survey questions were reviewed and approved by an expert attending physician and resident physicians to ensure they adequately captured the premise of the training sessions.

Confidence assessment

Participants rated their confidence in managing four types of injuries, including concussions, ankle injuries, knee injuries, and fractures, using a 10-point Likert scale (1 = not at all confident, 10 = extremely confident). These injuries were selected based on their high prevalence in adolescent athletes, the core demographic served by HLs during field assignments [16]. For analysis and cohort comparison, confidence scores were converted into percentages, allowing for standardized interpretation.

Knowledge assessment

Clinical knowledge was evaluated through four open-ended questions about the content shared with both training groups. The questions, listed in Table 1, assessed recognition of injury-specific diagnostic tests and management principles.

**Table 1:** Clinical Knowledge Questions and Answers This table lists the questions and answers included in the clinical knowledge assessment.

Question	Answer
What do the anterior drawer and talar tilt tests assess in ankle injuries?	The ankle anterior drawer test evaluates the anterior talofibular ligament (ATFL); the talar tilt test evaluates the calcaneofibular ligament (CFL).
What does the anterior drawer test assess in knee injuries?	The knee anterior drawer test evaluates the anterior cruciate ligament (ACL).
What is the best initial method to treat shin splints?	Rest, Ice, Compress, Elevate (RICE)
What is the first thing you should do when assisting an athlete with heat stroke?	Remove the athlete from the source of heat.

Participants who were unsure were instructed to respond with “idk.” Responses were scored as correct (1 point) or incorrect (0 points), including “idk”. The percentage of correct responses for each participant was calculated and averaged across the cohort to determine the cohort's overall performance.

Statistical evaluation and comparative analysis

Survey data from both training cohorts were analyzed to assess within-group improvements and between-group differences. Paired samples t-tests were used to compare pre- and post-training scores within each individual cohort. Independent samples t-tests were used to compare the change scores, which were the amount of improvement observed between the two cohorts for each assessment category. Key outcome measures included changes in HLs’ confidence in managing athletic injuries, improvement in their objective understanding of sports-related conditions, and a comparison of the degree of improvement experienced by each cohort.

In the simulation-based training cohort, we included an additional targeted question to evaluate the perceived value of resident physician involvement. This item captured participants' perceptions of the educational impact of residents' facilitation of clinical stations and simulated real-world patient handoffs.

## Results

The comparative analysis revealed statistically and educationally meaningful differences, underscoring the enhanced impact of hands-on, experiential learning on early clinical training. HLs in both training groups (didactic and simulation) demonstrated statistically significant improvements in self-reported confidence levels across all injury types evaluated. In the lecture-based (didactic) cohort, the mean increase in confidence ranged from 2.13 (ankle) to 2.81 (concussion), all with significant improvement (p < 0.001) as seen in Table 2. The simulation-based training cohort exhibited even greater improvements, with mean differences ranging from 3.03 (ankle) to 3.65 (knee), all of which were statistically significant (p < 0.001). Notably, compared to the didactic group, the simulation group consistently reported higher post-training confidence across all injury domains.

**Table 2:** Pre- and Post-Test Comparisons of Health Leader Confidence in Assessing Different Injuries Didactic and simulation training formats produced significant improvements in confidence. Std Dev: standard deviation Paired samples t-test computed with Microsoft Excel. ***statistically significant with a p-value <0.001

Assessment	Cohort	Sample Size	Pre-Test Mean	Pre-Test Std Dev	Post-Test Mean	Post-Test Std Dev	Mean Difference	T Value	P-Value
Concussion	Didactic	16	5.06	1.84	7.88	1.09	2.81	7.34	<0.001***
	Simulation	31	4.71	2.16	8.16	1.16	3.45	7.83	<0.001***
Knee	Didactic	16	5.31	1.74	7.56	1.55	2.25	5.38	<0.001***
	Simulation	31	4.51	2.22	8.16	1.34	3.65	7.82	<0.001***
Ankle	Didactic	16	5.81	1.87	7.94	1.12	2.13	5.44	<0.001***
	Simulation	31	4.94	2.24	7.97	1.3	3.03	6.53	<0.001***
Fracture	Didactic	16	4.81	2.01	7.44	1.15	2.63	6.32	<0.001***
	Simulation	31	4.61	2.42	7.71	1.6	3.1	5.95	<0.001***

Furthermore, simulation-based instruction led to significantly greater confidence gains compared to the lecture-based format. The average confidence improvement in the simulation group was 33.06%, compared to 24.53% in the didactic group (mean difference = 8.53%, p = <0.02*), as shown in Table 3. Although the simulation group also showed a larger average gain in knowledge scores (62.1% vs. 31.25%), this difference did not reach statistical significance (mean difference = 30.85%, p = 0.165), likely due to the greater variability in scores, as reflected by the higher standard deviation in the didactic group (32.7% vs. 18.9%).

**Table 3:** Comparison of Training Format on Improving a Health Leader’s Confidence and Knowledge Simulation training improved confidence significantly more than the didactic approach. Percent improved = the average amount of improvement each cohort demonstrated between pre- and post-tests of confidence and knowledge. Independent samples t-test computed with Microsoft Excel. *Statistically significant with a p-value <0.05.

Category	Training Group	Sample Size	Average (Percent Improved)	Standard Deviation	Mean Difference	T Value	P-Value
Confidence Improvement	Didactic	16	24.53%	3.20%	8.53%	3.94	0.02*
	Simulation	31	33.06%	2.91%			
Knowledge Improvement	Didactic	16	31.25%	32.7%	30.85%	1.63	0.153
	Simulation	31	62.10%	18.9%			

**Figure 1:**
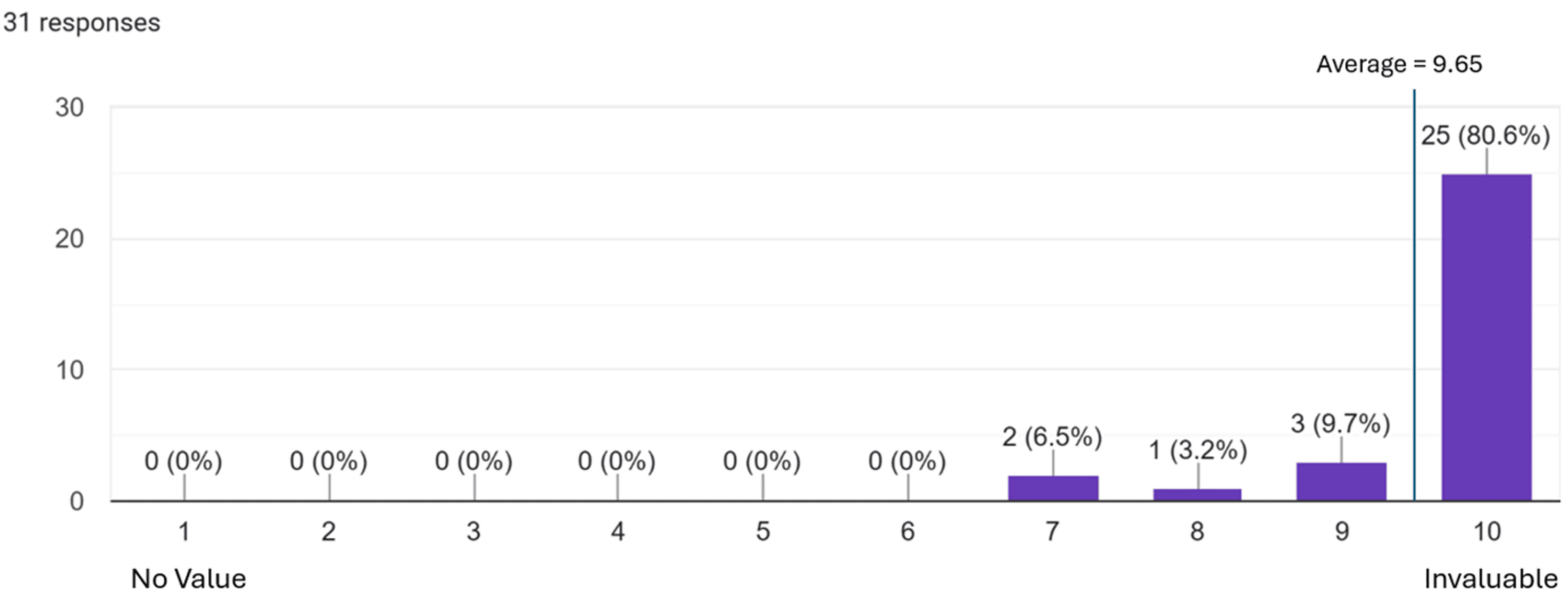
Value of Resident Physician Involvement Twenty-five (80.6%) trainees overwhelmingly rated resident physician involvement as invaluable.

A dedicated survey item assessed the added value of resident physician involvement in the simulation-based training. Respondents overwhelmingly affirmed its importance, with 25 (80.6%) participants rating resident participation a 10/10 or “invaluable.” The average rating for the value of resident involvement was 9.65 out of 10. This result is shown in Figure 1. In addition to providing expert demonstrations and real-time instruction, residents contributed to a more authentic learning environment by simulating the experience of handing off an injured patient to an on-call physician. Their presence enhanced the clinical realism of the scenarios and reinforced essential communication and decision-making skills critical to sideline care.

## Discussion

Interpretation of findings

Our findings contribute to the growing body of evidence supporting SBME as a promising approach for undergraduate training. In our study, participants in the simulation group demonstrated greater improvements in clinical competence, particularly in self-reported confidence, following simulation exercises. These results are consistent with prior studies in which students trained with SBME outperformed those receiving lectures alone in various performance metrics [17,18,19]. Specifically, HLs in the simulation group showed significantly greater confidence gains across all injury categories assessed: +3.45 in concussion management, +3.65 in knee injuries, +3.03 in ankle injuries, and +3.10 in fracture care (all p < 0.001). By contrast, the lecture-based cohort experienced smaller but still significant increases, ranging from +2.13 to +2.81. Overall, the simulation cohort demonstrated a 33.06% average confidence increase compared to 24.53% in the lecture group, a statistically significant difference of 8.53% (p = 0.02). These findings align with studies such as Angarita et al. (2019), where students who received simulation-based breast exam training performed 88% of the required OSCE steps compared to only 28% in the lecture group (p < 0.00001) [17].

Knowledge acquisition and cognitive retention

In terms of knowledge acquisition, our results showed a positive trend favoring simulation, though the difference did not reach statistical significance. Simulation-trained HLs improved their quiz scores by an average of 62.1% compared to 31.25% in the lecture group (p = 0.153). While this finding must be interpreted cautiously due to the p-value and sample size limitations, the observed trend aligns with existing literature suggesting SBME enhances long-term knowledge retention and skill acquisition. For instance, Alluri et al. (2024) reported that students in simulation and lecture groups initially performed similarly, but only the simulation group retained their performance on delayed testing [18]. This supports the idea that repeated, contextual engagement in SBME can promote more durable learning. Furthermore, the authentic nature of our simulation, combined with immediate feedback from resident instructors, likely contributed to learner engagement and cognitive reinforcement. Previous studies have shown that simulation enhances confidence, preparedness, and learner satisfaction, which echoes our observed outcomes [19].

Theoretical framework and educational design

Our simulation design incorporated principles from situated learning theory and Kirkpatrick’s four-level model. Situated learning emphasizes acquiring knowledge through authentic, socially interactive contexts [20], which our sideline scenarios simulated by placing learners in realistic athletic-event environments within underserved communities. Kirkpatrick’s model evaluates training across four levels: reaction, learning, behavior, and results [21]. At the reaction level, 80.6% of participants rated resident involvement a perfect 10, underscoring its perceived value. At the learning and behavior levels, HLs demonstrated improved fluency in communication and examination tasks through SBAR handoffs and injury assessments. These improvements reflect how SBME fosters deliberate practice and skill-building in realistic but controlled environments. This is particularly valuable for students with limited clinical exposure in high-pressure settings. Simulation sessions designed around real-world challenges, such as concussion and musculoskeletal injury assessments, helped bridge the gap between theoretical knowledge and practical competence. The observed gains in confidence and skill application suggest meaningful educational impact across multiple levels of Kirkpatrick’s model.

Equity, feasibility, and peer-driven learning

Importantly, this initiative is both student-led and implemented in a resource-limited, underserved setting, yet it yielded results comparable to larger institutional programs. This model exemplifies an inreach strategy, a locally driven and equity-oriented approach that directly addresses healthcare disparities within the community. Student-led SBME, when thoughtfully designed, can achieve outcomes on par with traditional faculty-led instruction. For example, Christiansen et al. (2023) demonstrated that peer-led debriefings were as effective as faculty-led ones [22], and during the COVID-19 pandemic, Ekert et al. showed that student-delivered simulation significantly increased provider confidence (p < 0.001) [23]. Our findings affirm the potential of peer-driven, community-engaged simulation as both feasible and impactful. This dual-purpose model expands access to high-quality training while empowering students to educate one another.

Limitations and future directions

To our knowledge, this is one of the first studies to compare SBME and lecture-based education in a student-volunteer, sideline medicine setting serving underserved urban populations. While our findings suggest that simulation-based training offers meaningful benefits, particularly in learner confidence, the study is limited by modest sample size, unequal group distribution, and lack of long-term outcome data. The observed knowledge improvements, while promising, did not reach statistical significance and should be interpreted with caution. Future studies with larger, balanced cohorts and delayed post-intervention assessments are needed to confirm and expand on these early findings. We also plan to follow participants across upcoming athletic seasons to assess skill retention, real-world performance, and continued engagement. Including these longitudinal metrics in future analyses will help clarify the long-term impact of this model.

## Conclusions

This study shows that simulation-based training leads to greater improvements in medical students’ confidence for volunteer sideline medical roles, demonstrating statistically significant gains (p = 0.02). Although knowledge improvements were noted in the simulation group, these did not reach statistical significance (p = 0.153), and conclusions regarding knowledge gains should be interpreted with caution. Realistic mock scenarios, immediate physician feedback, and structured communication using SBAR created a practical training environment closely mirroring real field conditions, fostering deeper engagement and skill application.

Importantly, this project illustrates how a student-led outreach program can integrate high-yield clinical training within an inreach framework that addresses local healthcare disparities by focusing resources on nearby, underserved communities. The positive impact of resident involvement underscores the value of near-peer teaching in enhancing educational quality. While the volunteer-based sample and absence of direct real-world performance assessments may limit the immediate generalizability of these findings, they provide a valuable foundation for future research. As medical education continues to emphasize earlier clinical exposure and community engagement, programs like DPSHC represent a promising and scalable model that effectively combines hands-on learning with meaningful service. Building on these results, further studies examining long-term outcomes and objective clinical performance will help to fully realize the potential of simulation-based training in enhancing student preparedness and patient care in underserved settings.
